# Late health effects and changes in lifestyle factors after cancer in childhood with and without subsequent second primary cancers – the KiKme case-control study

**DOI:** 10.3389/fonc.2022.1037276

**Published:** 2022-10-17

**Authors:** Lara Kim Brackmann, Ronja Foraita, Heike Schwarz, Danuta Galetzka, Sebastian Zahnreich, Thomas Hankeln, Markus Löbrich, Alicia Poplawski, Desiree Grabow, Maria Blettner, Heinz Schmidberger, Manuela Marron

**Affiliations:** ^1^ Epidemiological Methods and Etiological Research, Leibniz Institute for Prevention Research and Epidemiology – BIPS, Bremen, Germany; ^2^ Faculty of Mathematics and Computer Science, University of Bremen, Bremen, Germany; ^3^ Biometry and Data Management, Leibniz Institute for Prevention Research and Epidemiology – BIPS, Bremen, Germany; ^4^ Department of Radiation Oncology and Radiation Therapy, University Medical Center of the Johannes Gutenberg University, Mainz, Germany; ^5^ Institute of Organismic and Molecular Evolution, Molecular Genetics and Genome Analysis, Johannes Gutenberg University, Mainz, Germany; ^6^ Radiation Biology and deoxyribonucleic acid (DNA) Repair, Technical University of Darmstadt, Darmstadt, Germany; ^7^ Institute of Medical Biostatistics, Epidemiology and Informatics, University Medical Center of the Johannes Gutenberg University, Mainz, Germany; ^8^ German Childhood Cancer Registry, Institute for Medical Biostatistics, Epidemiology and Informatics, University Medical Center of the Johannes Gutenberg University, Mainz, Germany

**Keywords:** childhood cancer survivors (CCS), body mass index - BMI, physical activity, diet, alcohol, smoking, thyroid disease, lipid metabolism

## Abstract

**Background:**

Improved treatments for childhood cancer result in a growing number of long-term childhood cancer survivors (CCS). The diagnosis and the prevalence of comorbidities may, however, influence their lifestyle later in life. Nonetheless, little is known about differences in late effects between CCS of a first primary neoplasm (FPN) in childhood and subsequent second primary neoplasms (SPN) and their impact on lifestyle. Therefore, we aim to investigate associations between the occurrence of FPN or SPN and various diseases and lifestyle in the later life of CCS.

**Methods:**

CCS of SPN (n=101) or FPN (n=340) and cancer-free controls (n=150) were matched by age and sex, and CCS additionally by year and entity of FPN. All participants completed a self-administered questionnaire on anthropometric and socio-economic factors, medical history, health status, and lifestyle. Mean time between FPN diagnosis and interview was 27.3 years for SPN and 26.2 years for FPN CCS. To confirm results from others and to generate new hypotheses on late effects of childhood cancer as well as CCS´ lifestyles, generalized linear mixed models were applied.

**Results:**

CCS were found to suffer more likely from diseases compared to cancer-free controls. In detail, associations with cancer status were observed for hypercholesterinemia and thyroid diseases. Moreover, CCS were more likely to take regular medication compared to controls. A similar association was observed for CCS of SPN compared to CCS of FPN. In contrast to controls, CCS rarely exercise more than 5 hours per week, consumed fewer soft and alcoholic drinks, and were less likely to be current, former, or passive smokers. Additionally, they were less likely overweight or obese. All other exploratory analyses performed on cardiovascular, chronic lung, inflammatory bone, allergic, and infectious diseases, as well as on a calculated health-score revealed no association with tumor status.

**Conclusion:**

CCS were more affected by pathologic conditions and may consequently take more medication, particularly among CCS of SPN. The observed higher disease burden is likely related to the received cancer therapy. To reduce the burden of long-term adverse health effects in CCS, improving cancer therapies should therefore be in focus of research in this area.

## Introduction

Childhood cancer is a rare condition with about 400,000 new cases worldwide in the age group from 0 to 19 years (1). To date, there are only few established risk factors for the onset of childhood cancer. Besides rare genetic disorders (2–4), exposure to ionizing radiation and specific chemical substances (5) are known to be involved in the development of childhood cancer. Even though treatment options had significantly improved over the past decades, childhood cancer remains a leading cause of morbidity and mortality in this age group (6). As a result of the enhanced therapeutic efficacy, the number of childhood cancer survivors (CCS), and especially long-term CCS, has increased over time (7, 8). However, the incidence in survival is accompanied by adverse late health effects, which are associated with cancer therapy in childhood (9–14). Approximately three out of four CCS suffer from chronic health conditions 30 years after their cancer diagnosis (15), and about 8% of survivors of cancer under the age of 15 in Germany are diagnosed with a second primary malignancy within 30 years of their first diagnosis in Germany (16). In addition, cardiovascular diseases occurring at young ages have become a major cause of morbidity and mortality in CCS (8, 17, 18). In a large American cohort of CCS it has been shown that a reduction in radiation exposure to the heart during therapy reduces long-term effects in adulthood (7). However, cancer therapy may not only directly modulate the risk of cardiovascular diseases itself but also *via* modulation of other risk factors for cardiovascular diseases such as hypertension, dyslipidemia, and diabetes (19–21). Results from the aforementioned survivor cohort showed that former childhood cancer patients were more likely to take medication for the classical risk factors of cardiovascular diseases (hypertension, dyslipidemia, and diabetes) than their healthy siblings (21). It has been proven that, in addition to physical health, the mental health status of adult CCS is also affected by the comprehensive experience in childhood (22–25). The onset of mental health diseases in former childhood cancer patients could be accompanied by alcohol consumption (26, 27). Although former childhood cancer patients are less likely to be heavy drinkers compared to control groups in general (27–29), especially CCS that are living without a partner tend to consume alcohol more often than married ones (28). In addition, alcohol consumption may be associated with the education level, stress, and physical as well as social functionality (28, 29). Along with alcohol consumption, especially with heavy drinking habits, former childhood cancer patients are more likely to smoke (30). However, in the absence of alcohol consumption, the majority of CCS smoked less overall than the control groups (29, 31–33). Both, smoking and drinking are established risk factors for the development of several adverse health effects. Because of the toxins and mutagens present in alcohol, tobacco, and its additives, their use may have additive or even synergistic effects on preexisting risk factors for adverse health effects in CCS (30, 34, 35).

Therefore, the primary endpoint of this study is to provide a comprehensive overview of parameters on clinical information as well as the participants´ lifestyle and to confirm known associations between childhood cancer and late effects within the nested case-control study KiKme (German: “*Krebserkrankungen im Kindesalter und molekulare Epidemiologie*”, English: “*Cancer in childhood and molecular epidemiology*”) (36). As a secondary endpoint, we aim to generate new hypotheses on novel associations between cancer status, especially regarding CCS of at least one second primary neoplasm (SPN), and adverse late effects of childhood cancer therapies as well as lifestyle parameters in the framework of the KiKme study. To achieve these aims, we will compare CCS with cancer-free controls as well as CCS with FPN with CCS with SPN.

## Materials and methods

### Study design and participants

All participants of this study were recruited within the population-based nested case-control study KiKme. Detailed recruiting strategies and information on the general data collection were described elsewhere (36). Briefly, the study population consists of 441 CCS, registered at the German Childhood Cancer Registry, and 150 cancer-free controls. In the study, we differentiate between CCS with a first primary neoplasm (FPN, n=340) and CCS with SPN (n=101). FPN CCS were used as cancer controls and were therefore matched to participating SPN CCS by age, sex, cancer site, year of diagnosis, and age at diagnosis to participating SPN CCS. Cancer-free controls were recruited at the Department of Orthopedics and Trauma Surgery at the Johannes Gutenberg-University in Mainz (Germany) and matched by sex and age to the SPN and FPN participants.

### Data collection

All information for this study was collected using a questionnaire that was self-completed by the participants. The questionnaire included information on anthropometric and socio-economic factors, medical history, health status, and lifestyle parameters. As anthropometric factors, weight and height were requested. Based on this information, the Body Mass Index (BMI) was calculated by dividing weight in kilograms by height squared in meters (kg/m^2^). Normal weight was defined as BMI between 18.5 and <25 kg/m^2^, overweight as BMI ≥25 kg/m^2^, and obesity as BMI ≥30 kg/m^2^ according to the WHO and NIH standards (37). The educational level of the study participants was assessed using the International Standard Classification of Education (ISCED) (38). To assess their medical history and health status, participants were asked whether they take any regular medication and whether they have been diagnosed with one of the following diseases: diabetes, hypercholesterolemia, hypertension, lung diseases such as asthma or bronchitis, hay fever, inflammatory joint or vertebral diseases including arthrosis and rheumatism, neurodermatitis, heart attack, stroke, thyroid diseases, Epstein-Barr virus infections, HIV, Hepatitis, or any other severe disease. Additionally, age at diagnosis for each of the applicable diseases was requested. Smoking and drinking habits were requested, along with consumption of soft drinks, water, coffee, and other drinks, using scaled information per day or week. Using this information, alcoholic beverages per day and pack-years were calculated. In addition, participants were asked about their extent of regular physical activities. Based on all data collected, we then created a score that should depict the general health status of the participants. A maximum of 8 points could be achieved in this health score and the awarding of points were made up as follows: 2 or fewer diseases (1 point), 3 or more diseases (0 points); normal weight defined as BMI between 18.5-30 (1 point), BMI below 18.8 or higher than 30 (0 points); high ISCED defined as upper secondary education or above (1 point), lower secondary and primary education (0 points); never smoker (1 point), current or former smoker (0 points); less than one alcoholic beverage per day (1 point), one or more alcoholic beverages per day (0 points); no consumption of soft drinks (1 point), consumption of soft drinks (0 points); 5 or more hours of physical activity per week (1 point), less than 5 hours physical activity per week (0 points); currently employed or self-employed (1 point), incapacitated or retired (0 points). For the calculation of the health score, at least 4 of the 8 items had to be answered by the participant. If less than 4 items were answered, the health score was set to missing. The total number of points of each participant was then divided by the number of variables that were not missing and the score was divided into 3 categories (<0.75 points, 0.75 points, > 0.75 points).

### Statistical analysis

Descriptive analyses were conducted to calculate sample characteristics regarding anthropometric and socio-economic factors, medical history, health status, and lifestyle parameters stratified by cancer status (SPN, FPN, and cancer-free controls). Summary statistics were provided in frequency (N) and proportions (%).

Generalized linear mixed models (GLMM) were applied to estimate the associations between categorical and dichotomous outcome variables, the late effects, with cancer status (SPN vs. FPN) and with case-control status (CCS vs. cancer-free controls) using odds ratios (OR) and 95% confidence intervals (CIs). We treated the matched groups as random effects and ‘age’ and ‘year of birth’ as fixed effects in all models to improve matching efficiency for the variable ‘age at recruitment’ within the specified 5-year period. Additional adjustment variables for each GLMM were identified *via* Directed Acyclic Graphs (DAGs) that were carefully developed based on prior knowledge using DAGitty 3.0^1^ (39) (see Supplementary File 1). All health- and lifestyle-related outcomes that were collected *via* the self-administered questionnaire were taken into account for analyses unless they had less than 5% expression per characteristic across all groups were excluded from the analyses. All statistical analyses for this publication were performed using SAS 9.3 (SAS Institute Inc., Cary, North Carolina, USA).

## Results

### Study characteristics

The study sample consists of 101 SPN, 340 FPN, and 150 cancer-free controls with 51% females and 49% males (**Table 1**). However, only the 554 study participants (94%) with sufficient information from self-administered questionnaires could be analyzed depending on the set inclusion criteria for these analyses. The mean age at interview among them was 35.14 years (standard deviation (SD): 7.14; range: 19.90-51.40 years) for CCS of SPN, 34.84 years (SD: 7.68; range: 19.60-54.50 years) for CCS of FPN, and 28.91 years (SD: 7.32; range: 18.70-48.20 years) for cancer-free controls. On average, at the time of the interview, the first cancer diagnosis had occurred 27.26 years (SD: 6.90; range: 5.00-38.00 years) earlier in CCS of SPN and 26.24 years (SD: 6.93; range: 4.00-39.00 years) earlier in CCS of FPN. A total of 90% of study participants indicated their ethnicity as Caucasian. While the CCS included in this study came from all over Germany, the majority of cancer-free controls came from Rhineland-Palatinate due to recruitment at the University Hospital in Mainz. Further characteristics of the study participants including detailed information on health and lifestyle are summarized in **Table 1** and **Table 2**.

**Table 1:** Distribution of cases (SPN and FPN) and controls (CO) of the KiKme study.

	Total (N=591)		SPN (N=101)		FPN (N=340)		CO (N=150)	
	n	%	n	%	n	%	n	%
**Questinnaire available**								
yes	**554**	**94%**	85	84%	325	96%	144	96%
no	**37**	**6%**	16	16%	15	4%	6	4%
**Sex**								
female	**301**	**51%**	50	50%	189	56%	62	41%
male	**290**	**49%**	51	50%	151	44%	88	59%
**Age at interview**	**591**							
<25 years	**100**	**17%**	9	9%	37	11%	54	36%
25-29 years	**106**	**18%**	14	14%	55	16%	37	25%
30-34 years	**113**	**19%**	17	17%	76	22%	20	13%
35-39 years	**111**	**19%**	21	21%	70	21%	20	13%
40 years or more	**124**	**21%**	24	24%	87	26%	13	9%
no questionnaire	**37**	**6%**	16	16%	15	4%	6	4%
**Ethnicity**								
Caucasian	**533**	**90%**	84	83%	312	92%	137	91%
other ethnicity^1^	**20**	**3%**	0	0%	12	4%	7	5%
no information	**38**	**6%**	17	17%	16	5%	6	4%
**State**								
Lower Saxony	**36**	**6%**	8	8%	28	8%	0	0%
North Rhine-Westphalia	**101**	**17%**	18	18%	80	24%	3	2%
Hesse	**60**	**10%**	7	7%	29	9%	24	16%
Rhineland-Palatinate	**127**	**21%**	0	0%	18	5%	109	73%
Baden-Wuerttemberg	**60**	**10%**	18	18%	41	12%	1	1%
Bavaria	**83**	**14%**	19	19%	64	19%	0	0%
every other German state^2^	**76**	**13%**	15	15%	59	17%	2	1%
outside Germany	**6**	**1%**	0	0%	6	2%	0	0%
no information	**42**	**7%**	16	16%	15	4%	11	7%
**Height**								
< 160cm	**51**	**9%**	10	10%	36	11%	5	3%
160 - <170cm	**190**	**32%**	35	35%	124	36%	31	21%
170 - <180cm	**166**	**28%**	21	21%	95	28%	50	33%
180 - <190cm	**113**	**19%**	15	15%	60	18%	38	25%
190cm or more	**33**	**6%**	4	4%	9	3%	20	13%
no information	**38**	**6%**	16	16%	16	5%	6	4%
**Weight**								
< 60kg	**102**	**17%**	20	20%	70	21%	12	8%
60 - <70kg	**134**	**23%**	19	19%	84	25%	31	21%
70 - <80kg	**98**	**17%**	17	17%	58	17%	23	15%
80 - <90kg	**99**	**17%**	14	14%	54	16%	31	21%
90 - <100kg	**61**	**10%**	10	10%	27	8%	24	16%
100kg or more	**55**	**9%**	4	4%	28	8%	23	15%
no information	**42**	**7%**	17	17%	19	6%	6	4%
**Body Mass Index**								
normal weight	**297**	**50%**	47	47%	178	52%	72	48%
overweight	**168**	**28%**	28	28%	93	27%	47	31%
obese	**84**	**14%**	9	9%	50	15%	25	17%
no information	**42**	**7%**	17	17%	19	6%	6	4%
**Physical activity (hours per week)**								
0 hours	**248**	**42%**	42	42%	149	44%	57	38%
1 - 2 hours	**84**	**14%**	18	18%	53	16%	13	9%
3 - 4 hours	**102**	**17%**	13	13%	67	20%	22	15%
5 hours or more	**110**	**19%**	8	8%	53	16%	49	33%
no information	**47**	**8%**	20	20%	18	5%	9	6%
**Consumption of soft drinks per day**								
0	**105**	**18%**	26	26%	63	19%	16	11%
<1	**298**	**50%**	43	43%	165	49%	90	60%
1 or more	**92**	**16%**	10	10%	60	18%	22	15%
no information	**96**	**16%**	22	22%	52	15%	22	15%
**Alcoholic beverages per day**								
0	**124**	**21%**	29	29%	70	21%	25	17%
<1	**359**	**61%**	46	46%	222	65%	91	61%
1 or more	**48**	**8%**	5	5%	24	7%	19	13%
no information	**60**	**10%**	21	21%	24	7%	15	10%
**Smoking status**								
never smoked	**348**	**59%**	57	56%	224	66%	67	45%
former smoker	**82**	**14%**	14	14%	44	13%	24	16%
current smoker	122	21%	14	14%	56	16%	52	35%
no information	**39**	**7%**	16	16%	16	5%	7	5%
**Pack years**								
never smoked	**348**	**59%**	57	56%	224	66%	67	45%
<5	**76**	**13%**	10	10%	47	14%	19	13%
5 or more	**103**	**17%**	18	18%	46	14%	39	26%
no information	**64**	**11%**	16	16%	23	7%	25	17%
**Passive smoker**								
yes	**70**	**12%**	10	10%	31	9%	29	19%
no	**464**	79%	75	74%	281	83%	108	72%
no information	**57**	**10%**	16	16%	28	8%	13	9%
**Living situation**								
living alone	**144**	**24%**	23	23%	75	22%	46	31%
living without a partner, with children	**23**	**4%**	6	6%	14	4%	3	2%
living with a partner, without children	**142**	**24%**	20	20%	85	25%	37	25%
living with a partner and children	**149**	**25%**	24	24%	102	30%	23	15%
living with parents	**67**	**11%**	11	11%	34	10%	22	15%
living in a shared apartment	**22**	**4%**	1	1%	11	3%	10	7%
other living situation^3^	**4**	**1%**	0	0%	2	1%	2	1%
no information	**40**	**7%**	16	16%	17	5%	7	5%
**Main occupation**								
still in training	**94**	**16%**	10	10%	38	11%	46	31%
working full time	**300**	**51%**	44	44%	185	54%	71	47%
working part time	**77**	**13%**	12	12%	55	16%	10	7%
housewife/-man	**21**	**4%**	4	4%	14	4%	3	2%
job seeking	**19**	**3%**	5	5%	8	2%	6	4%
pensioner or unemployable	**18**	**3%**	6	6%	10	3%	2	1%
other occupation^4^	**17**	**3%**	2	2%	12	4%	3	2%
no information	**45**	**8%**	18	18%	18	5%	9	6%
**Highest school degree**								
Volks-/Hauptschulabschluss	**76**	**13%**	14	14%	44	13%	18	12%
Realschulabschluss/Mittlere Reife	**152**	**26%**	27	27%	91	27%	34	23%
Fachhochschulreife	**75**	**13%**	4	4%	45	13%	26	17%
Abitur/Hochschulreife	**241**	**41%**	38	38%	140	41%	63	42%
no graduation (yet)	**5**	**1%**	2	2%	3	1%	0	0%
no information	**42**	**7%**	16	16%	17	5%	9	6%
**Highest vocational education**								
completed apprenticeship	**196**	**33%**	28	28%	123	36%	45	30%
graduated from vocational/business school	**72**	**12%**	12	12%	46	14%	14	9%
graduated from a technical college	**47**	**8%**	8	8%	26	8%	13	9%
graduated from college	**151**	**26%**	24	24%	102	30%	25	17%
no graduation (yet)	**71**	**12%**	11	11%	20	6%	40	27%
no information	**54**	**9%**	18	18%	23	7%	13	9%
**International Standard Classification of Education**								
no graduation (yet)	**5**	**1%**	2	2%	3	1%	0	0%
Sek I	**25**	**4%**	6	6%	6	2%	13	9%
Sek II	**322**	**54%**	45	45%	187	55%	90	60%
academic or equal	**198**	**34%**	32	32%	128	38%	38	25%
no information	**41**	**7%**	16	16%	16	5%	9	6%
**Children**								
0	**191**	**32%**	36	36%	117	34%	38	25%
1	**71**	**12%**	13	13%	50	15%	8	5%
2	**63**	**11%**	10	10%	45	13%	8	5%
3 or more	**31**	**5%**	6	6%	19	6%	6	4%
no information	**235**	**40%**	36	36%	109	32%	90	60%

cancer-free control (CO), first primary neoplasm (FPN), second primary neoplasm (SPN).

^1^Asian (total = 1%), Latino (1%), Caucasian/Latino (1%), Black (0.3%), North African (0.3%), Caucasian/Asian (0.3%), Caucasian/Black (0.2%).

^2^Schleswig-Holstein (total = 2%), Hamburg (2%), Berlin (2%), Saxony (2%), Bremen (1%), Saarland (1%), Brandenburg (1%), Mecklenburg-Western Pomerania (1%), Thuringia (1%), Saxony-Anhalt (0.3%).

^3^With family and partner (total = 0.3%), with siblings (0.3%).

^4^Parental leave (total = 1%), sheltered workshop (0.3%), internship/volunteering (0.3%), self-employed (0.2%), marginal employment (0.2%), other, not specified (1%). Significant values were printed in bold.

**Table 2:** Distribution of variables on health status of cases (SPN and FPN) and controls (CO).

	Total (N=591)		SPN (N=101)		FPN (N=340)		CO (N=150)	
	n	%	n	%	n	%	n	%
**Therapy for FPN**								
no cancer therapy	**3**	**1%**	1	1%	2	1%	0	0%
radiotherapy	**4**	**1%**	3	3%	1	0%	0	0%
chemotherapy	**102**	**17%**	15	15%	87	26%	0	0%
radio- and chemotherapy	**209**	**35%**	46	46%	163	48%	0	0%
only other cancer therapy (e.g., operation)	**1**	**0%**	0	0%	1	0%	0	0%
radio- and other cancer therapy	**1**	**0%**	0	0%	1	0%	0	0%
chemo- and other cancer therapy	**18**	**3%**	4	4%	14	4%	0	0%
radio-, chemo- and other cancer therapy	**63**	**11%**	13	13%	50	15%	0	0%
no information	**190**	**32%**	19	19%	21	6%	150	100%
**Family with possible Li-Fraumeni syndrome**								
yes	**73**	**12%**	21	21%	52	15%	0	0%
no	**483**	**82%**	64	63%	273	80%	146	97%
no information	**35**	**6%**	16	16%	15	4%	4	3%
**Regular medication**								
yes	**298**	**50%**	65	64%	180	53%	53	35%
no	**247**	**42%**	19	19%	140	41%	88	59%
no information	**46**	**8%**	17	17%	20	6%	9	6%
**Any diseases**								
yes	**379**	**64%**	70	69%	241	71%	68	45%
no	**174**	**29%**	15	15%	84	25%	75	50%
no information	**38**	**6%**	16	16%	15	4%	7	5%
**Number of diseases**								
0	**174**	**29%**	15	15%	84	25%	75	50%
1	**182**	**31%**	28	28%	116	34%	38	25%
2	**114**	**19%**	26	26%	68	20%	20	13%
3	**49**	**8%**	10	10%	29	9%	10	7%
4 or more	**34**	**6%**	6	6%	28	8%	0	0%
no information	**38**	**6%**	16	16%	15	4%	7	5%
**Diabetes**								
yes	**26**	**4%**	4	4%	22	6%	0	0%
no	**519**	**88%**	79	78%	301	89%	139	93%
no information	**46**	**8%**	18	18%	17	5%	11	7%
**Thyroid diseases (without cancer)**								
yes	**140**	**24%**	18	18%	117	34%	5	3%
no	**384**	**65%**	42	42%	208	61%	134	89%
no information	**67**	**11%**	41	41%	15	4%	11	7%
**Hypercholesterolemia**								
yes	**66**	**11%**	15	15%	48	14%	3	2%
no	**475**	**80%**	67	66%	274	81%	134	89%
no information	**50**	**8%**	19	19%	18	5%	13	9%
**Cardiovascular diseases (hypertension, heart attack, or stroke)**								
yes	**69**	**12%**	13	13%	46	14%	10	7%
no	**469**	**79%**	69	68%	271	80%	129	86%
no information	**53**	**9%**	19	19%	23	7%	11	7%
**Hypertension**								
yes	**61**	**10%**	11	11%	40	12%	10	7%
no	**482**	**82%**	71	70%	282	83%	129	86%
no information	**48**	**8%**	19	19%	18	5%	11	7%
**Heart attack**								
yes	**4**	**1%**	0	0%	4	1%	0	0%
no	**540**	**91%**	83	82%	318	94%	139	93%
no information	**47**	**8%**	18	18%	18	5%	11	7%
**Stroke**								
yes	**6**	**1%**	2	2%	4	1%	0	0%
no	**534**	**90%**	81	80%	314	92%	139	93%
no information	**51**	**9%**	18	18%	22	6%	11	7%
**Chronic lung diseases**								
yes	**64**	**11%**	**9**	9%	35	10%	20	13%
no	**481**	**81%**	**73**	72%	287	84%	121	81%
no information	**46**	**8%**	19	19%	18	5%	9	6%
**Inflammatory bone diseases**								
yes	**66**	**11%**	9	9%	42	12%	15	10%
no	**476**	**81%**	72	71%	280	82%	124	83%
no information	**49**	**8%**	20	20%	18	5%	11	7%
**Allergic diseases (hay fever or neurodermatitis)**								
yes	**156**	**26%**	24	**24%**	91	27%	41	27%
no	**387**	**65%**	59	58%	228	67%	100	67%
no information	**48**	**8%**	18	18%	21	6%	9	6%
Hay fever								
yes	**120**	**20%**	17	17%	72	21%	31	21%
no	**423**	**72%**	66	65%	248	73%	109	73%
no information	**48**	**8%**	18	18%	20	6%	10	7%
**Neurodermatitis**								
yes	**53**	**9%**	8	8%	32	9%	13	9%
no	**492**	**83%**	75	74%	290	85%	127	85%
no information	**46**	**8%**	18	18%	18	5%	10	7%
**Infections (hepatitis, Epstein-Barr virus, or HIV)**								
yes	**79**	**13%**	17	17%	51	15%	11	7%
no	**441**	**75%**	66	65%	272	80%	103	69%
no information	**71**	**12%**	18	18%	17	5%	36	24%
Hepatitis								
yes	**11**	**2%**	4	4%	6	2%	1	1%
no	**508**	**86%**	79	78%	316	93%	113	75%
no information	**72**	**12%**	18	18%	18	5%	36	24%
**Epstein-Barr virus**								
yes	**68**	**12%**	13	13%	45	13%	10	7%
no	**453**	**77%**	70	69%	279	82%	104	69%
no information	**70**	**12%**	18	18%	16	5%	36	24%
**HIV**								
yes	**1**	**0%**	1	1%	0	0%	0	0%
no	**518**	**88%**	82	81%	322	95%	114	76%
no information	**72**	**12%**	18	18%	18	5%	36	24%
**Health score^1^**								
< 0.75 points	**276**	**47%**	44	44%	152	45%	80	53%
0.75 points	**154**	**26%**	23	23%	100	29%	31	21%
> 0.75 points	**123**	**21%**	18	18%	72	21%	33	22%
no information	**38**	**6%**	16	16%	16	5%	6	4%

cancer-free control (CO), first primary neoplasm (FPN), second primary neoplasm (SPN).

^1^Score includes the following variables: number of diseases: 0-2 (1 point (p.)), 3 or more (0 p.); Body Mass Index: <18.5 (0 p.), 18.5-30 (1 p.), >30 (0 p.); International Standard Classification of Education (ISCED): Sek II or academic (1 p.), no graduation or Sek I (0 p.); smoking status: never (1 p.), former or current (0 p.); alcoholic beverages/day: <1 (1 p.), 1 or more (0 p.); consumption of softdrinks: no (1 p.), yes (0 p.); hours of physical activity/week: 5 hours or more (1 p.), 0-4 hours (0 p.); current occupation: occupied (1 p.), unemployable or pensioner (0 p.). At least 4 items of the score have to be answered. To account for missing values, the sum score was divided by the number of answered variables. Significant values were printed in bold.

### Association between cancer status and lifestyle factors

In our study population, we observed that CCS were less likely to be overweight (unadjusted (unadj.): OR=0.59 (95%CI 0.36;0.96), adjusted (adj).: OR=0.56 (95%CI 0.34; 0.92)) or obese (unadj.: OR=0.48 (95%CI 0.27, 0.87), adj.: OR=0.51 (95%CI 0.27, 0.96)) than cancer-free controls (**Table 3**). In terms of physical activity, former cancer patients were less likely to exercise more than 5 hours per week than cancer-free controls (unadj.: OR=0.47 (95%CI 0.28; 0.82), adj.: OR =0.42 (95% CI 0.24, 0.73)). In addition, SPN and FPN subjects consumed fewer sugar-sweetened beverages than cancer-free controls. This decreased consumption was found to be statistically significant when consumption of less than one drink per day was compared to consumption of no drink (unadj.: OR=0.45 (95%CI 0.24; 0.86), adj.: OR=0.43 (95% CI 0.22; 0.82)). The comparison between CCS with SPN and FPN also showed that CCS with SPN drink more than one sweetened beverage per day less often than CCS with FPN only (unadj.: OR=0.41 (95%CI 0.18; 0.95), adj.: OR=0.42 (95% CI 0.18; 1.00)). We also observed differences in alcohol consumption per day. Here, an association for the comparison between more than one drink and no drink per day could be observed between CCS and cancer-free controls (unadj.: OR=0.34 (95%CI 0.14; 0.80), adj.: OR=0.30 (95% CI 0.12, 0.73)). In addition, a suggested association for the consumption of less than 1 alcoholic drink per day was found in the comparison between the two groups of CCS. However, this association was only significant in the unadjusted model and, when further adjustment variables were included, this result just exceeded the significance limit (SPN versus FPN unadj.: OR=0.46 (95%CI 0.27; 0.79), adj.: OR=0.55 (95% CI 0.29, 1.02)). While a conducted sensitivity analysis, comparing only leukemia CCS to cancer-free controls, also reveals a significant result in the consumption of more than one drink compared to no drink when comparing CCS and cancer-free controls as well as in the consumption of less than one drink compared to no drink when comparing CCS of SPN to CCS of FPN, a sensitivity analysis for CCS of lymphoma did not show any association (**Supplementary Table 1**). An even stronger association for the comparison of the consumption of more than one drink and no drink per day was found in a stratified analysis including only participants living together with a partner (adj. OR=0.12 (0.03; 0.57). It was also found that CCS were less likely to be current (unadj.: OR=0.45 (95%CI 0.25; 0.82), adj.: OR=0.43 (95%CI 0.24; 0.79)), former (unadj.: OR=0.28 (95%CI 0.17, 0.49), adj.: OR=0.25 (95%CI 0.15; 0.44)), or passive smokers (unadj.: OR=0.47 (95% CI 0.26; 0.85, **Table 3** and **Table 4**) than cancer-free controls. This effect was consistent in all conducted sensitivity analyses and, again, even more pronounced in participants living together with a partner (Supplementary File 1). In the conducted sensitivity analysis including only CCS with lymphoma, moreover, CCS of SPN were found to be more often passive smokers than CCS of FPN (adj. OR=3.83 (1.04; 14.2, **Supplementary Table 1**). However, such an association was neither found in other stratified analyses nor in the analysis including all study participants. For the models on smoking, no further adjustments were necessary according to the DAGs. Based on the smoking status, it was also found that the number of pack years consumed was lower among CCS than among cancer-free controls (unadj.: OR=0.21 (95%CI 0.12; 0.39), adj.: OR =0.17 (95%CI 0.09; 0.33), **Table 3**).

**Table 3:** Multinomial logistic regression on cancer status and risk of late effects^1^.

Cancer status	n	%	n	%	n	%	n	%	n	%	n	%	OR (95% CI)	OR (95% CI)	OR (95% CI)	OR (95% CI)	OR (95% CI)	OR (95% CI)
													adjusted for matchinggroup, birth year, and age at interview			adjusted for matchinggroup, birth year, age at interview, and other variables		
**Body Mass Index**																		
** **	**total (N=591)**		**missings (N=42)**		**normal weight (N=297)**		**overweight (N=168)**		**obesity (N=84)**				**overweight vs. normal weight**	**obesity vs. normal weight**		**overweight vs. normal weight**	**obesity vs. normal weight**	
CO	**150**	**25%**	6	14%	72	24%	47	28%	25	30%			Ref.	Ref.		Ref.^2^	Ref.^2^	
FPN and SPN	**441**	**75%**	36	86%	225	76%	121	72%	59	70%			**0.587 (0.359; 0.96)**	**0.479 (0.265; 0.866)**		**0.561 (0.344; 0.915)**	**0.506 (0.268; 0.958)**	
	** **	** **																
FPN	**340**	**77%**	19	53%	178	79%	93	77%	50	85%			Ref.	Ref.		Ref.^3^	Ref.^3^	
SPN	**101**	**23%**	17	47%	47	21%	28	23%	9	15%			1.144 (0.659; 1.986)	0.686 (0.305; 1.543)		1.183 (0.659; 2.126)	0.532 (0.198; 1.431)	
**Physical activity (hours per week)**																		
	**total (N=591)**		**missings (N=47)**		**0 hours (N=248)**		**1-2 hours (N=84)**		**3-4 hours (N=102)**		**5+ hours (N=110)**		**1-2 hours vs. 0 hours**	**3-4 hours vs. 0 hours**	**5+ hours vs. 0 hours**	**1-2 hours vs. 0 hours**	**3-4 hours vs. 0 hours**	**5+ hours vs. 0 hours**
CO	**150**	**25%**	9	19%	57	23%	13	15%	22	22%	49	45%	Ref.	Ref.	Ref.	Ref.^4^	Ref.^4^	Ref.^4^
FPN and SPN	**441**	**75%**	38	81%	191	77%	71	85%	80	78%	61	55%	1.684 (0.805; 3.524)	1.194 (0.648; 2.199)	**0.474 (0.276; 0.815)**	1.491 (0.691; 3.214)	1.089 (0.577; 2.055)	**0.416 (0.236; 0.734)**
	** **	** **											** **			** **		
FPN	**340**	**77%**	18	47%	149	78%	53	75%	67	84%	53	87%	Ref.	Ref.	Ref.	Ref.^4^	Ref.^4^	Ref.^4^
SPN	**101**	**23%**	20	53%	42	22%	18	25%	13	16%	8	13%	1.19 (0.614; 2.306)	0.632 (0.304; 1.312)	0.507 (0.211; 1.222)	1.261 (0.63; 2.525)	0.671 (0.315; 1.43)	0.544 (0.22; 1.344)
**Consumption of soft drinks per day**																		
** **	**total (N=591)**		**missing (N=96)**		**0 (N=105)**		**<1 (N=298)**		**1+ (N=92)**		** **		**<1 vs. 0**	**1+ vs. 0**		**<1 vs. 0**	**1+ vs. 0**	** **
CO	**150**	**25%**	22	23%	16	15%	90	30%	22	24%			Ref.	Ref.		Ref.^4^	Ref.^4^	
FPN and SPN	**441**	**75%**	74	77%	89	85%	208	70%	70	76%			**0.453 (0.24; 0.856)**	0.734 (0.324; 1.664)		**0.426 (0.222; 0.819)**	0.699 (0.303; 1.612)	
	** **	** **											** **			** **		
FPN	**340**	**77%**	52	70%	63	71%	165	79%	60	86%			Ref.	Ref.		Ref.^4^	Ref.^4^	
SPN	**101**	**23%**	22	30%	26	29%	43	21%	10	14%			0.65 (0.363; 1.166)	**0.41 (0.176; 0.953)**		0.676 (0.378; 1.212)	0.423 (0.179; 1)	
**Alcoholic beverages per day**																		
	**total (N=591)**		**missings (N=60)**		**0 (N=124)**		**<1 (N=359)**		**1+ (N=48)**				**<1 vs. 0**	**1+ vs. 0**		**<1 vs. 0**	**1+ vs. 0**	
CO	**150**	**25%**	15	25%	25	20%	91	25%	19	40%			Ref.	Ref.		Ref.^2^	Ref.^2^	
FPN and SPN	**441**	**75%**	45	75%	99	80%	268	75%	29	60%			0.715 (0.421; 1.216)	**0.34 (0.144; 0.8)**		0.663 (0.376; 1.171)	**0.296 (0.121; 0.727)**	
	** **	** **											** **			** **		
FPN	**340**	**77%**	24	53%	70	71%	222	83%	24	83%			Ref.	Ref.		Ref.^3^	Ref.^3^	
SPN	**101**	**23%**	21	47%	29	29%	46	17%	5	17%			**0.459 (0.267; 0.788)**	0.449 (0.136; 1.482)		0.546 (0.294; 1.015)	0.475 (0.13; 1.74)	
**Smoking status**																		
** **	**total (N=591)**		**missing (N=39)**		**never (N=348)**		**former (N=82)**		**current (N=122)**		** **		**former vs. never**	**current vs. never**	** **	**former vs. never**	**current vs. never**	** **
CO	**150**	**25%**	7	18%	67	19%	24	29%	52	43%			Ref.	Ref.		Ref.^4^	Ref.^4^	
FPN and SPN	**441**	**75%**	32	82%	281	81%	58	71%	70	57%			**0.283 (0.165; 0.486)**	**0.453 (0.251; 0.816)**	** **	**0.252 (0.146; 0.435)**	**0.43 (0.235; 0.787)**	** **
	** **	** **																
FPN	**340**	**77%**	16	50%	224	80%	44	76%	56	80%			Ref.	Ref.		Ref.^5^	Ref.^5^	
SPN	**101**	**23%**	16	50%	57	20%	14	24%	14	20%			0.866 (0.43; 1.742)	1.334 (0.68; 2.617)		0.99 (0.448; 2.186)	1.435 (0.704; 2.925)	
**Pack years**																		
	**total (N=591)**		**missings (N=64)**		**never smoked (N=348)**		**<5 (N=76)**		**5+ (N=103)**		** **		**<5 vs. never smoked**	**5+ vs. never smoked**	** **	**<5 vs. never smoked**	**5+ vs. never smoked**	** **
CO	**150**	**25%**	25	39%	67	19%	19	25%	39	38%			Ref.	Ref.		Ref.^4^	Ref.^4^	
FPN and SPN	**441**	**75%**	39	61%	281	81%	57	75%	64	62%			0.814 (0.435; 1.523)	**0.213 (0.115; 0.393)**	** **	0.837 (0.436; 1.608)	**0.173 (0.09; 0.333)**	** **
	** **	** **											** **			** **		
FPN	**340**	**77%**	23	59%	224	80%	47	82%	46	72%			Ref.	Ref.		Ref.^5^	Ref.^5^	
SPN	**101**	**23%**	16	41%	57	20%	10	18%	18	28%			0.928 (0.437; 1.97)	1.571 (0.796; 3.1)	** **	1.092 (0.455; 2.622)	1.547 (0.682; 3.509)	** **

Adjustment variables were selected using directed acyclic graphs.

Confidence interval (CI), cancer-free control (CO), first primary neoplasm (FPN), International Standard Classification for Education (ISCED), odds ratio (OR), second primary neoplasm (SPN).

^1^Missing values are shown but not included in the analysis.

^2^Additionally adjusted for ISCED and ethnicity.

^3^Additionally adjusted for ISCED, ethnicity, and therapy of FPN.

^4^Additionally adjusted for ISCED.

^5^Additionally adjusted for ISCED and therapy of FPN. Significant values were printed in bold.

**Table 4:** Comparison of serum IL-4, PGE2 and AGEs in each group.

Cancer status	n	%	n	%	n	%	n	%	OR (95% CI)	OR (95% CI)
									adjusted for matchinggroup, birth year, and age at interview	adjusted for matchinggroup, birth year, age at interview, and other variables
**Passive smoker**										
	**total (N=591)**		**missing (N=57)**		**no (N=464)**		**yes (N=70)**		**yes vs. no**	**yes vs. no**
CO	**150**	**25%**	13	23%	108	23%	29	41%	Ref.	Ref.^2^
FPN and SPN	**441**	**75%**	44	77%	356	77%	41	59%	**0.471 (0.261; 0.849)**	**0.471 (0.261; 0.849)**
	** **	** **							** **	** **
FPN	**340**	**77%**	28	64%	281	79%	31	76%	Ref.	Ref.^3^
SPN	**101**	**23%**	16	36%	75	21%	10	24%	1.206 (0.55; 2.646)	1.19 (0.465; 3.048)
**Regular medication**										
	**total (N=591)**		**missing (N=46)**		**no (N=247)**		**yes (N=298)**		**yes vs. no**	**yes vs. no**
CO	**150**	**25%**	9	20%	88	36%	53	18%	Ref.	Ref.^2^
FPN and SPN	**441**	**75%**	37	80%	159	64%	245	82%	**2.301 (1.352; 3.917)**	**2.301 (1.352; 3.917)**
	** **	** **							** **	** **
FPN	**340**	**77%**	20	54%	140	88%	180	73%	Ref.	Ref.^3^
SPN	**101**	**23%**	17	46%	19	12%	65	27%	2.938 (0.77; 11.212)	**2.527 (1.013; 6.304)**
**Any disease**										
	**total (N=591)**		**missing (N=70)**		**no (N=175)**		**yes (N=379)**		**yes vs. no**	**yes vs. no**
CO	**150**	**25%**	7	18%	75	43%	68	18%	Ref.	Ref.^4^
FPN and SPN	**441**	**75%**	31	82%	99	57%	311	82%	**3.549 (2.095; 6.014)**	**3.322 (1.952; 5.652)**
	** **	** **								
FPN	**340**	**77%**	15	48%	84	85%	241	77%	Ref.	Ref.^5^
SPN	**101**	**23%**	16	52%	15	15%	70	23%	1.903 (0.883; 4.101)	1.531 (0.735; 3.189)
**Thyroid diseases (without cancer)**										
	**total (N=591)**		**missing (N=67)**		**no (N=384)**		**yes (N=140)**		**yes vs. no**	**yes vs. no**
CO	**150**	**25%**	11	16%	134	35%	5	4%	Ref.	Ref.^4^
FPN and SPN	**441**	**75%**	56	84%	250	65%	135	96%	**15.007 (5.636; 39.954)**	**14.703 (5.488; 39.387)**
	** **	** **							** **	** **
FPN	**340**	**77%**	15	27%	208	83%	117	87%	Ref.	Ref.^5^
SPN	**101**	**23%**	41	73%	42	17%	18	13%	0.702 (0.362; 1.361)	0.658 (0.309; 1.402)
**Hypercholesterolemia**										
	**total (N=591)**		**missing (N=50)**		**no (N=475)**		**yes (N=66)**		**yes vs. no**	**yes vs. no**
CO	**150**	**25%**	13	26%	134	28%	3	5%	Ref.	Ref.^4^
FPN and SPN	**441**	**75%**	37	74%	341	72%	63	95%	**6.836 (2.028; 23.04)**	**7.205 (2.126; 24.424)**
	** **	** **							** **	** **
FPN	**340**	**77%**	18	49%	274	80%	48	76%	Ref.	Ref.^5^
SPN	**101**	**23%**	19	51%	67	20%	15	24%	1.215 (0.622; 2.37)	1.29 (<0.001; >999.99)
**Cardiovascular diseases (hypertension, heart attack (SPN=0, FPN=4, CO=0), or stroke (SPN=2, FPN=4, CO=0))**										
	**total (N=591)**		**missing (N=53)**		**no (N=469)**		**yes (N=69)**		**yes vs. no**	**yes vs. no**
CO	**150**	**25%**	11	21%	129	28%	10	14%	Ref.	Ref.^4^
FPN and SPN	**441**	**75%**	42	79%	340	72%	59	86%	1.765 (0.836; 3.725)	1.487 (0.693; 3.192)
	** **	** **							** **	** **
FPN	**340**	**77%**	23	55%	271	80%	46	78%	Ref.	Ref.^5^
SPN	**101**	**23%**	19	45%	69	20%	13	22%	1.194 (0.59; 2.417)	1.006 (0.433; 2.336)
**Hypertension**										
	**total (N=591)**		**missing (N=48)**		**no (N=482)**		**yes (N=61)**		**yes vs. no**	**yes vs. no**
CO	**150**	**25%**	11	23%	129	27%	10	16%	Ref.	Ref.^4^
FPN and SPN	**441**	**75%**	37	77%	353	73%	51	84%	1.493 (0.69; 3.229)	1.283 (0.582; 2.83)
	** **	** **							** **	** **
FPN	**340**	**77%**	18	49%	282	80%	40	78%	Ref.	Ref.^5^
SPN	**101**	**23%**	19	51%	71	20%	11	22%	1.141 (0.529; 2.465)	0.942 (0.379; 2.343)
**Chronic lung diseases**										
	**total (N=591)**		**missing (N=46)**		**no (N=481)**		**yes (N=64)**		**yes vs. no**	**yes vs. no**
CO	**150**	**25%**	9	20%	121	25%	20	31%	Ref.	Ref.^4^
FPN and SPN	**441**	**75%**	37	80%	360	75%	44	69%	0.741 (0.394; 1.392)	0.598 (0.305; 1.176)
	** **	** **							** **	** **
FPN	**340**	**77%**	18	49%	287	80%	35	80%	Ref.	Ref.^5^
SPN	**101**	**23%**	19	51%	73	20%	9	20%	1.082 (0.485; 2.416)	0.767 (0.275; 2.137)
**Inflammatory bone diseases**										
	**total (N=591)**		**missing (N=49)**		**no (N=476)**		**yes (N=66)**		**yes vs. no**	**yes vs. no**
CO	**150**	**25%**	11	22%	124	26%	15	23%	Ref.	Ref.^4^
FPN and SPN	**441**	**75%**	38	78%	352	74%	51	77%	0.737 (0.383; 1.421)	0.776 (0.397; 1.516)
	** **	** **								
FPN	**340**	**77%**	18	47%	280	80%	42	82%	Ref.	Ref.^5^
SPN	**101**	**23%**	20	53%	72	20%	9	18%	0.877 (0.401; 1.919)	0.79 (0.313; 1.996)
**Allergic diseases (hay fever or neurodermatitis)**										
	**total (N=591)**		**missing (N=48)**		**no (N=387)**		**yes (N=156)**		**yes vs. no**	**yes vs. no**
CO	**150**	**25%**	9	19%	100	26%	41	26%	Ref.	Ref.^4^
FPN and SPN	**441**	**75%**	39	81%	287	74%	115	74%	0.916 (0.572; 1.465)	0.83 (0.509; 1.352)
	** **	** **							** **	** **
FPN	**340**	**77%**	21	54%	228	79%	91	79%	Ref.	Ref.^5^
SPN	**101**	**23%**	18	46%	59	21%	24	21%	1.1 (0.63; 1.921)	0.993 (0.495; 1.994)
**Hay fever**										
	**total (N=591)**		**missing (N=48)**		**no (N=423)**		**yes (N=120)**		**yes vs. no**	**yes vs. no**
CO	**150**	**25%**	10	21%	109	26%	31	26%	Ref.	Ref.^4^
FPN and SPN	**441**	**75%**	38	79%	314	74%	89	74%	0.898 (0.539; 1.494)	0.817 (0.481; 1.388)
	** **	** **							** **	** **
FPN	**340**	**77%**	20	53%	248	79%	72	81%	Ref.	Ref.^5^
SPN	**101**	**23%**	18	47%	66	21%	17	19%	0.944 (0.51; 1.747)	0.88 (0.093; 8.297)
**Neurodermatitis**										
	**total (N=591)**		**missing (N=46)**		**no (N=492)**		**yes (N=53)**		**yes vs. no**	**yes vs. no**
CO	**150**	**25%**	10	22%	127	26%	13	25%	Ref.	Ref.^4^
FPN and SPN	**441**	**75%**	36	78%	365	74%	40	75%	1.264 (0.609; 2.622)	1.225 (0.577; 2.601)
	** **	** **							** **	** **
FPN	**340**	**77%**	18	50%	290	79%	32	80%	Ref.	Ref.^5^
SPN	**101**	**23%**	18	50%	75	21%	8	20%	1.046 (0.445; 2.463)	0.816 (0.267; 2.493)
**Infections (hepatitis (SPN=4, FPN=6, CO=1), Epstein-Barr virus, or HIV (SPN=1, FPN=0, CO=0))**										
	**total (N=591)**		**missing (N=71)**		**no (N=441)**		**yes (N=79)**		**yes vs. no**	**yes vs. no**
CO	**150**	**25%**	36	51%	103	23%	11	14%	Ref.	Ref.^4^
FPN and SPN	**441**	**75%**	35	49%	338	77%	68	86%	1.757 (0.874; 3.533)	1.831 (0.9; 3.725)
										
FPN	**340**	**77%**	17	49%	272	80%	51	75%	Ref.	Ref.^5^
SPN	**101**	**23%**	18	51%	66	20%	17	25%	1.306 (0.701; 2.43)	1.43 (0.689; 2.97)
**Epstein-Barr virus infection**										
	**total (N=591)**		**missing (N=70)**		**no (N=453)**		**yes (N=68)**		**yes vs. no**	**yes vs. no**
CO	**150**	**25%**	36	51%	104	23%	10	15%	Ref.	Ref.^4^
FPN and SPN	**441**	**75%**	34	49%	349	77%	58	85%	1.637 (0.791; 3.392)	1.689 (0.805; 3.544)
	** **	** **								
FPN	**340**	**77%**	16	47%	279	80%	45	78%	Ref.	Ref.^5^
SPN	**101**	**23%**	18	53%	70	20%	13	22%	1.039 (0.529; 2.039)	1.16 (0.529; 2.543)

Adjustment variables were selected using directed acyclic graphs.

Confidence interval (CI), cancer-free control (CO), first primary neoplasm (FPN), human immunodeficiency virus (HIV), International Standard Classification for Education (ISCED), odds ratio (OR), second primary neoplasm (SPN).

^1^Missing values are shown but not included in the analysis.

^2^DAGs identified no additional adjustment variables for this model.

^3^Additionally adjusted for therapy of FPN.

^4^Additionally adjusted for families with possible Li-Fraumeni syndrome.

^5^Additionally adjusted for therapy of FPN and families with possible Li-Fraumeni syndrome. Significant values were printed in bold.

### Association between cancer status and late adverse health effects

Overall, the CCS in our study had more serious illnesses (cancer excluded) than the cancer-free controls subjects (unadj.: OR =3.55 (95%CI 2.10, 6.01), adj.: OR=3.32 (95%CI 1.95, 5.65), **Table 4**). In the analysis of individual diseases, it was found that CCS suffer more frequently from thyroid diseases (unadj.: OR=15.01 (95%CI 5.64; 39.95), adj.: OR=14.70 (95%CI 5.49, 39.39)) and hypercholesterolemia (unadj.: OR=6.84 (95%CI 2.03, 23.04), adj.: OR=7.21 (95%CI 2.13; 24.42)) compared to cancer-free controls. In addition, it was found that CCS were more likely to take regular medication than cancer-free controls (unadj.: OR=2.30 (95%CI 1.35; 3.92), no further adjustment according to DAGs necessary). Here, the adjusted comparison between the CCS groups with SPN and with FPN only additionally showed that in our study population CCS of SPN took more medication than those with FPN only (OR=2.53 (95%CI 1.01; 6,30)). All other explorative conducted analyses on cardiovascular, chronic lung, inflammatory bone, allergic, and infectious diseases did not show any associations.

### Association between cancer status and a calculated overall health score

The majority of the study participants (n=276, 47%) achieved a score below 0.75 points in our health score (**Table 2**). About a quarter (n=154, 26%) of the participants achieved exactly 0.75 points. Only 123 participants (21%) reached the highest category with a score above 0.75 points. In addition to the subjects without a questionnaire, one additional participant had to be excluded from the health score analysis since the required 4 answers for the calculation of the score were not given. The multinomial logistic regression on cancer status and calculated health score did not show any associations (**Table 5**).

**Table 5:** Multinomial logistic regression on cancer status and calculated health score^1^.

Cancer status	n	%	n	%	n	%	n	%	n	%	OR (95% CI)	OR (95% CI)	OR (95% CI)	OR (95% CI)
											adjusted for matchinggroup, birth year, and age at interview		adjusted for matchinggroup, birth year, and age at interview^2^	
**Health score (max. 1 point (p.); all participants)**														
	**total (N=591)**		**missings (N=37)**		**< 0.75 p. (N=276)**		**0.75 p. (N=154)**		**> 0.75 p. (N=124)**		**< 0.75 p. vs. > 0.75 p.**	**0.75 p. vs. > 0.75 p.**	**< 0.75 p. vs. > 0.75 p.**	**0.75 p. vs. > 0.75 p.**
CO	**150**	**25%**	6	16%	80	29%	31	20%	33	27%	Ref.	Ref.	Ref.	Ref.
FPN and SPN	**441**	**75%**	31	84%	196	71%	123	80%	91	73%	0.745 (0.445; 1.247)	1.616 (0.871; 2.998)	0.687 (0.4; 1.181)	1.425 (0.744; 2.731)
														
FPN	**340**	**77%**	15	48%	152	78%	100	81%	73	80%	Ref.	Ref.	Ref.	Ref.
SPN	**101**	**23%**	16	52%	44	22%	23	19%	18	20%	1.112 (0.595; 2.078)	0.913 (0.456; 1.827)	1.06 (0.563; 1.995)	0.78 (0.382; 1.592)
**Health score (max. 1 p.; females)**														
	**total (N=301)**		**missings (N=14)**		**< 0.75 p. (N=134)**		**0.75 p. (N=93)**		**> 0.75 p. (N=60)**		**< 0.75 p. vs. > 0.75 p.**	**0.75 p. vs. > 0.75 p.**	**< 0.75 p. vs. > 0.75 p.**	**0.75 p. vs. > 0.75 p.**
CO	**62**	**21%**	3	21%	31	23%	17	18%	11	18%	Ref.	Ref.	Ref.	Ref.
FPN and SPN	**239**	**79%**	11	79%	103	77%	76	82%	49	82%	0.701 (0.308; 1.598)	1.088 (0.442; 2.681)	0.597 (0.247; 1.443)	1.103 (0.412; 2.95)
														
FPN	**189**	**79%**	5	45%	80	78%	62	82%	42	86%	Ref.	Ref.	Ref.	Ref.
SPN	**50**	**21%**	6	55%	23	22%	14	18%	7	14%	1.681 (0.655; 4.312)	1.397 (0.514; 3.794)	1.623 (0.624; 4.22)	1.318 (0.479; 3.631)
**Health score (max. 1 p.; males)**														
	**total (N=290)**		**missings (N=23)**		**< 0.75 p. (N=142)**		**0.75 p. (N=61)**		**> 0.75 p. (N=64)**		**< 0.75 p. vs. > 0.75 p.**	**0.75 p. vs. > 0.75 p.**	**< 0.75 p. vs. > 0.75 p.**	**0.75 p. vs. > 0.75 p.**
CO	**88**	**30%**	3	13%	49	35%	14	23%	22	34%	Ref.	Ref.	Ref.	Ref.
FPN and SPN	**202**	**70%**	20	87%	93	65%	47	77%	42	66%	0.812 (0.404; 1.631)	1.839 (0.759; 4.46)	0.679 (0.263; 1.752)	1.274 (0.491; 3.308)
														
FPN	**151**	**75%**	10	50%	72	77%	38	81%	31	74%	Ref.	Ref.	Ref.	Ref.
SPN	**51**	**25%**	10	50%	21	23%	9	19%	11	26%	0.765 (0.304; 1.926)	0.626 (0.204; 1.922)	0.739 (0.283; 1.929)	0.491 (0.152; 1.585)

Confidence interval (CI), cancer-free control (CO), first primary neoplasm (FPN), odds ratio (OR), second primary neoplasm (SPN).

^1^Score includes the following variables: number of diseases: 0-2 (1 point (p.)), 3 or more (0 p.); Body Mass Index: <18.5 (0 p.), 18.5-30 (1 p.), >30 (0 p.); International Standard Classification for Education (ISCED): Sek II or academic (1 p.), no graduation or Sek I (0 p.); smoking status: never (1 p.), former or current (0 p.); alcoholic beverages/day: <1 (1 p.), 1 or more (0 p.); consumption of soft drinks: no (1 p.), yes (0 p.); hours of physical activity/week: 5 hours or more (1 p.), 0-4 hours (0 p.); current occupation: occupied (1 p.), unemployable or pensioner (0 p.). At least 4 items of the score have to be answered. To account for missing values, the sum score was divided by the number of answered variables. Missing values are shown but not included in the analysis.

^2^All models were additionally adjusted for ethnicity and families with possible Li-Fraumeni syndrome. Adjustment variables were selected using directed acyclic graphs. Significant values were printed in bold.

## Discussion

Within the presented study, we attempted to complete the overall picture of the associations between childhood cancer and long-term effects on health and lifestyle factors. We show that CCS and cancer-free controls as well as CCS with and without subsequent SPN differ in terms of their health and lifestyle.

Although the CCS in our study were less likely to exercise extensively, they were less likely to be overweight or obese than cancer-free controls. Even if physical activity is known to reduce the risk of long-term adverse health outcomes after childhood cancer (40, 41) studies have shown that about 50% of CCS in western countries do not meet the recommended time of physical activity per day (42). This reduced time of physical activity among CCS might be due to poorer overall health. In this regard, a Swiss study showed that physical activity was reduced in CCS, particularly when they either had relapse or suffer from musculoskeletal or neurological disorders (42). Similar to our findings on weight status, a cohort of French leukemia survivors identified significant differences regarding the prevalence of metabolic syndrome and BMI between former acute lymphatic leukemia patients and cancer-free controls (43). In addition, they found differences in socio-economic status, education, occupation, and smoking habits. Whereas education was found to be an important adjustment variable in nearly all of our models and was therefore not investigated as an outcome, we were able to identify differences in smoking habits in our sample. Our CCS were less likely to be current, former, or passive smokers. This effect was even more pronounced in participants living together with a partner. Along with this healthier attitude towards smoking habits, there was also reduced consumption of alcohol among the CCS in our study sample. Here again, an even more reduced consumption was found in participants living in a partnership. Similar findings were also reported by Frobisher et al. (28), who reported that CCS living without a partner tend to consume alcohol more often than married ones. Moreover, Brinkman et al. (26) showed that CCS were less likely to be heavy or risky drinkers compared to their siblings. In general, the reduced alcohol consumption might be associated with the identified higher intake of regular medication in the CCS group, since the consumption of alcohol may interact with prescribed medications (44). However, this possible association was taken into account when creating the DAGs and here, it was shown that no further adjustment according to medication intake is necessary for the analysis of the association between cancer status and alcohol consumption. Regarding the increased regular intake of medication in the CCS group, our findings are in line with those from other research groups. Within the large American cohort of the Childhood Cancer Survivor Study it was found that CCS were more likely to take medication for hypertension, dyslipidemia, or diabetes compared to their siblings (21). However, at the same time, they were neither more often obese nor did they show more cardiovascular risk factors than their healthy siblings.

Although some other studies have found evidence of an association between cardiovascular diseases and childhood cancer (17–19), we have not observed such an association in our data. The absence of this known association can have various reasons: On the one hand, with a mean age of 32 years, we have a relatively young cohort (36). Moreover, we overall observed only very few cardiovascular events in our cohort, of which most were related to the presence of hypertension. However, due to the young cohort, in the course of the advancing observation period and the ongoing survival time, further cardiovascular diseases could occur. It can already be seen in our cohort that CCS more often suffer from disorders of the lipid metabolism, which is one of the main risk factors for the development of cardiovascular diseases (19, 45). Besides a higher prevalence of lipid metabolism disorders, the CCS of the KiKme study suffered from thyroid disorders significantly more frequently than cancer-free controls. Thyroid disorders are well-known adverse late health effects of cancer therapies and especially of cancer therapies in childhood (46). This known association between thyroid diseases and cancer therapy is well illustrated in our data as well since the strong effect observed when comparing CCS and cancer-free controls disappears when comparing SPN and FPN, both of whom received some type of cancer therapy.

Besides the confirmation of known associations in our data, we attempted to generate new hypotheses on novel associations between cancer status and adverse late health effects of childhood cancer as well as lifestyle parameters. Within the comparison between cases and controls, no new hypotheses could be generated. However, to the best of our knowledge, this study is the first one to investigate differences between CCS with a single diagnosis in childhood and CCS with multiple primary malignancies.

This comparison between CCS groups showed that CCS with SPN took more medication than those with FPN. This result complements the previously described hypothesis of reduced alcohol consumption with regular medication intake. It was also shown here, even if the result just exceeds the significance limit in the adjusted model (**Table 3**), that CCS with SPN, who take significantly more medication, drink alcoholic beverages (less than 1 drink/day compared to no drink) less frequently than CCS with FPN only. No difference was found for higher amounts (more than one drink/day) of alcohol per day. In addition, there was also a difference in the consumption of more than 1 soft drink per day, even if the significance limit in the adjusted model was also slightly exceeded here. Again, CCS with SPN were found to drink sugar-sweetened beverages less frequently than CCS with FPN (**Table 3**). This could be an indication of a more conscious lifestyle in general. These findings, however, need to be confirmed in larger studies.

Regarding the associations between cancer status and a calculated overall health score, other than expected from us, no difference was observed by cancer status in our study. This null result may be explained by the fact that, as our results showed, the cases might have a higher disease burden but live a healthier lifestyle overall. The cancer-free control group, on the other hand, appears to be healthier but have an unhealthier lifestyle. Thus, for the health score, which includes both health and lifestyle factors, the total scores obtained by cases and controls may annihilate.

### Strengths and limitations

Regarding strengths and limitations, this unique cohort of CCS with and without subsequent SPN and cancer-free controls is the first to carry out differentiated analyzes on cancer and late health effects as well as on differences in lifestyle, also at the level of different numbers of cancer diagnoses.

All information for the conducted analyses was self-reported by the participants and therefore might underlie a certain recall bias. However, by collecting self-reported data, we were able to get information on a large number of variables that enables us to extensively adjust our models. Moreover, we succeeded to collect not only information from the subjects themselves but also collected information on the family history of severe diseases, which allows us to adjust for familial predispositions to some extent.

As with all self-reported epidemiological studies, our study underlies an inherent survivor bias as only living patients could be recruited. Severe cases with high mortality (e.g. acute myeloid leukemia after acute lymphocytic leukemia or 2 diagnoses in quick succession) cannot be covered to a full extent by this study. Moreover, a selection bias cannot be ruled out in this study, as individuals with serious health problems may be less motivated to participate and the recruitment of cancer-free controls was regionally limited due to logistic reasons. Moreover, cancer-free controls were found to be slightly younger then participating CCS. In addition, the statistical power of the study is limited by the sample size. The number of available former childhood cancer patients was restricted by the number of CCS meeting the inclusion criteria that were registered at the German Childhood Cancer Registry. The sample size and the rather short follow-up time of CCS result in a small number of adverse health outcomes, especially for rare diseases such as heart attack, stroke, or serious infectious diseases. However, the number of late adverse health outcomes may increase during the further follow-up of our cohort. The cohort thus offers the possibility for extensive analyzes of late effects of childhood cancer in the future. With an increasing number of outcomes, more differentiated investigations, e.g., concerning the type, number, and localization of received therapies, can also be considered.

## Conclusion

Overall, a different general state of health and different health behaviors could be identified between CCS and cancer-free controls. Although CCS seem to have healthier lifestyles than cancer-free controls, including less soft drink and alcohol consumption as well as less tobacco smoking and lower body mass index, they are more likely to have serious illnesses. In detail, the results of this study conducted on German CCS and cancer-free controls, confirm that thyroid diseases without thyroid cancer and disorders of the lipid metabolism may be more common in CCS than in cancer-free controls. As a consequence of the higher disease burden, CCS, particularly those with SPN, may take more regular medication. In addition, CCS seem to be less physically active than cancer-free controls, which might be explained by their higher disease burden. The higher overall disease burden is likely related to previous cancer therapies. Based on these findings, research into improving cancer therapies and starting points for reducing long-term consequences should continue in the future. Moreover, we recommend that former childhood cancer patients be closely monitored by their treating physicians, not only with regard to cancer follow-up, but especially with regard to possible potential risk factors for the development of late adverse health effects. Here in particular, lipid metabolism disorders should be treated to prevent the development of cardiovascular disease. In addition, survivors should be encouraged to achieve the recommended time of physical activity, as this has been identified in the past as protective for the development of various adverse health outcomes in cancer survivors.

## Data availability statement

The datasets presented in this article are not readily available because of ethic and data protection reasons, but are available from the corresponding author on reasonable request. Requests to access the datasets should be directed to the Leibniz Institute for Prevention Research and Epidemiology – BIPS, Bremen, Germany.

## Ethics statement

The studies involving human participants were reviewed and approved by the Ethik-Kommission of the Landesärztekammer Rheinland-Pfalz, Mainz, Germany. The patients/participants provided their written informed consent to participate in this study.

## Authors contributions

MM is the principal investigator of the KiKme study and developed its design, which was implemented and monitored by MM and LB. DG supported the development of strategies for the recruitment of former childhood cancer patients. MM, LB, DGa, and SZ conducted the recruitment of the participants, which was organized and planned by MM and LB. MM, LB, and HS monitored the recruitment of cancer-free controls. LB, MM, RF, and AP developed the analysis pipelines for the project. HSz, DGa, SZ, TH, ML, AP, DGr, MB, and HS contributed to the writing process, which was initially prepared by LB, RF, and MM. All authors contributed to the article and approved the submitted version.

## Acknowledgments

The authors especially thank Claudia Spix from the German Childhood Cancer Registry for her assistance in developing strategies and materials for the recruitment of former childhood cancer patients with her long-standing experience in conducting register-based studies in Germany. In addition, we gratefully acknowledge the assistance from Franziska Himmelsbach, Cornelia Becker, Ilona Kerenyi, and Marianne Brömmel from the German Childhood Cancer Registry who identified, matched, and made the first contact with former childhood cancer patients. We are thankful for the central support of Patricia Sadre Fischer during the start of the recruitment and the excellent laboratory assistance of Ursula Disque-Kaiser and Iris Schmitt. We further thank Caine Lucas Grandt, Willempje Hummel-Bartenschlager, Claas Sontag, Katharina Musiolik, and Christin Goldbaum for their meticulous work on the databases. This work was supported by the Federal Ministry of Education and Research in Germany (Grants 02NUK016A, 02NUK042A, 02NUK042B, 02NUK042C, and 02NUK042D). The study is funded among other research projects as part of the ISIMEP (Intrinsic radiation sensitivity: Identification, mechanisms and epidemiology, principal investigator: MB) and the ISIBELA (Intrinsic radiation sensitivity: Identification of biological and epidemiological long-term effects, principal investigator: MB and HS) consortium.

## Conflict of interest

The authors declare that the research was conducted in the absence of any commercial or financial relationships that could be construed as a potential conflict of interest.

## Publisher’s note

All claims expressed in this article are solely those of the authors and do not necessarily represent those of their affiliated organizations, or those of the publisher, the editors and the reviewers. Any product that may be evaluated in this article, or claim that may be made by its manufacturer, is not guaranteed or endorsed by the publisher.

## Footnotes

1 http://www.dagitty.net/dags.html

## Abbreviations

Adj., Adjusted; BMI, Body mass index; CCS, Childhood cancer survivors; CI, Confidence interval; CO, Cancer-free controls; DAG, Directed Acyclic Graph; FPN, First primary neoplasm; GLMM, Generalized linear mixed model; ISCED, International Standard Classification of Education; KiKme, Krebs im Kindesalter und molekulare Epidemiologie; OR, Odds ratio; SD, Standard deviation; SPN, At least one second primary neoplasm; Unadj., Unadjusted.
