# Supplementary material for: Late health effects and changes in lifestyle factors after cancer in childhood with and without subsequent second primary cancers – the KiKme case-control study
Source: Front Oncol. 2022 Oct 17;12:1037276. doi: 10.3389/fonc.2022.1037276 (PMC9618813; doi:10.3389/fonc.2022.1037276)
Supplement: Supplementary file 1 [file DataSheet_1.docx]

**Supplementary File 1**

**Identification of adjustment variables using Directed Acyclic Graphs (DAGs)**

**Basic adjustment:**

Matching group, age at recruitment, birth year

**Identified adjustment variables using Directed Acyclic Graphs (DAGs):**

| **Outcome** | **Adjustment variables** |
| --- | --- |
| BMI | Ethnicity, ISCED, cancer therapy |
| Physical activity | ISCED |
| Soft drinks | ISCED |
| Alcohol | Ethnicity, ISCED, cancer therapy |
| Smoking | ISCED, cancer therapy |
| Pack years | ISCED, cancer therapy |
| Passive smoking | Cancer therapy |
| Medication | Cancer therapy |
| Comorbidities | Genetic family, cancer therapy |
| Health Score | Ethnicity, genetic family |


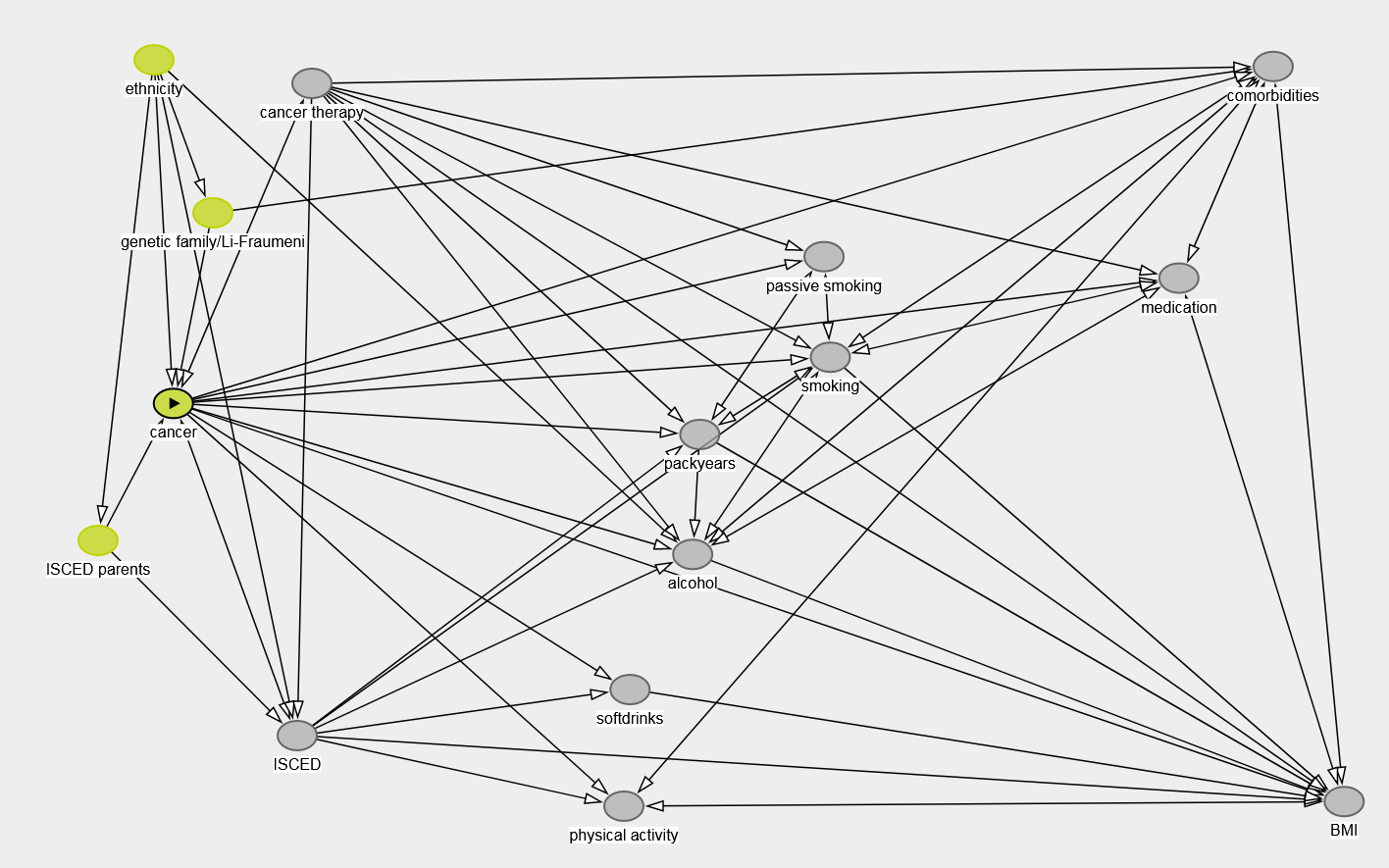


**Supplementary Figure 1:** DAG on the association between cancer in childhood and late adverse health effects as well as lifestyle parameters

**Code DAGs:**

dag {

"ISCED parents" [pos="-1.909,0.417"]

"cancer therapy" [pos="-1.367,-1.542"]

"genetic family/Li-Fraumeni" [pos="-1.618,-0.987"]

"passive smoking" [pos="-0.068,-0.799"]

"physical activity" [pos="-0.576,1.556"]

BMI [pos="1.251,1.536"]

ISCED [pos="-1.404,1.253"]

alcohol [pos="-0.401,0.477"]

cancer [exposure,pos="-1.718,-0.170"]

comorbidities [pos="1.071,-1.614"]

ethnicity [pos="-1.767,-1.642"]

medication [pos="0.832,-0.707"]

packyears [pos="-0.383,-0.037"]

smoking [pos="-0.052,-0.368"]

softdrinks [pos="-0.560,1.056"]

"ISCED parents" -> ISCED

"ISCED parents" -> cancer

"cancer therapy" -> "passive smoking"

"cancer therapy" -> BMI

"cancer therapy" -> ISCED

"cancer therapy" -> alcohol

"cancer therapy" -> comorbidities

"cancer therapy" -> medication

"cancer therapy" -> packyears

"cancer therapy" -> smoking

"cancer therapy" <-> cancer

"genetic family/Li-Fraumeni" -> cancer

"genetic family/Li-Fraumeni" -> comorbidities

"passive smoking" <-> packyears

"passive smoking" <-> smoking

"physical activity" <-> BMI

"physical activity" <-> comorbidities

BMI <-> comorbidities

BMI <-> medication

ISCED -> "physical activity"

ISCED -> BMI

ISCED -> alcohol

ISCED -> packyears

ISCED -> smoking

ISCED -> softdrinks

ISCED <-> cancer

alcohol -> BMI

alcohol <-> comorbidities

alcohol <-> medication

alcohol <-> smoking

cancer -> "passive smoking"

cancer -> "physical activity"

cancer -> BMI

cancer -> alcohol

cancer -> comorbidities

cancer -> medication

cancer -> packyears

cancer -> smoking

cancer -> softdrinks

comorbidities <-> medication

comorbidities <-> smoking

ethnicity -> "ISCED parents"

ethnicity -> "genetic family/Li-Fraumeni"

ethnicity -> ISCED

ethnicity -> alcohol

ethnicity -> cancer

medication <-> smoking

packyears -> BMI

packyears -> alcohol

packyears <-> smoking

smoking -> BMI

softdrinks -> BMI

}

**Supplementary File 2**

**Sensitivity analyses on lifestyle of childhood cancer survivors stratified by cancer site of first primary neoplasm and living situation**

**Supplementary Table 1:** Stratified adjusted logistic regression on cancer status and risk of late effects^1^

| **Cancer status** | **Leukemia** | | | **Lymphoma** | | | **Living with a partner** | | |
| --- | --- | --- | --- | --- | --- | --- | --- | --- | --- |
|  | **OR (95% CI)** | **OR (95% CI)** | **OR (95% CI)** | **OR (95% CI)** | **OR (95% CI)** | **OR (95% CI)** | **OR (95% CI)** | **OR (95% CI)** | **OR (95% CI)** |
| **Body Mass Index** | | | | | | | | | |
|  | **overweight vs. normal weight** | **obesity vs. normal weight** |  | **overweight vs. normal weight** | **obesity vs. normal weight** |  | **overweight vs. normal weight** | **obesity vs. normal weight** |  |
| CO | Ref.^2^ | Ref.^2^ |  | Ref.^2^ | Ref.^2^ |  | Ref.^2^ | Ref.^2^ |  |
| FPN and SPN | 0.565 (0.304; 1.052) | 0.563 (0.271; 1.171) |  | 0.603 (0.29; 1.256) | 0.388 (0.134; 1.121) |  | 0.466 (0.178; 1.223) | 0.473 (0.131; 1.717) |  |
|  |  |  |  |  |  |  |  |  |  |
| FPN | Ref.^3^ | Ref.^3^ |  | Ref.^3^ | Ref.^3^ |  | Ref.^3^ | Ref.^3^ |  |
| SPN | 0.954 (0.274; 3.319) | 0.71 (0.162; 3.116) |  | 2.35 (0.847; 6.523) | 1.393 (0.245; 7.914) |  | - ( -; -) | - ( -; -) |  |
| **Physical activity (hours per week)** | | | | | | | | | |
|  | **1-2 hours vs. 0 hours** | **3-4 hours vs. 0 hours** | **5+ hours vs. 0 hours** | **1-2 hours vs. 0 hours** | **3-4 hours vs. 0 hours** | **5+ hours vs. 0 hours** | **1-2 hours vs. 0 hours** | **3-4 hours vs. 0 hours** | **5+ hours vs. 0 hours** |
| CO | Ref.^4^ | Ref.^4^ | Ref.^4^ | Ref.^4^ | Ref.^4^ | Ref.^4^ | Ref.^4^ | Ref.^4^ | Ref.^4^ |
| FPN and SPN | - ( -; -) | - ( -; -) | - ( -; - ) | - ( -; -) | - ( -; -) | - ( -; - ) | - ( -; -) | - ( -; -) | - ( -; - ) |
|  |  |  |  |  |  |  |  |  |  |
| FPN | Ref.^4^ | Ref.^4^ | Ref.^4^ | Ref.^4^ | Ref.^4^ | Ref.^4^ | Ref.^4^ | Ref.^4^ | Ref.^4^ |
| SPN | - ( -; -) | - ( -; -) | - ( -; - ) | - ( -; -) | - ( -; -) | - ( -; - ) | - ( -; -) | - ( -; -) | - ( -; - ) |
| **Consumption of soft drinks per day** | | | | | | | | | |
|  | **<1 vs. 0** | **1+ vs. 0** |  | **<1 vs. 0** | **1+ vs. 0** |  | **<1 vs. 0** | **1+ vs. 0** |  |
| CO | Ref.^4^ | Ref.^4^ |  | Ref.^4^ | Ref.^4^ |  | Ref.^4^ | Ref.^4^ |  |
| FPN and SPN | **0.334 (0.156; 0.712)** | 0.467 (0.172; 1.268) |  | 0.537 (0.227; 1.268) | 1.807 (0.629; 5.194) |  | 0.474 (0.145; 1.54) | 0.636 (0.144; 2.804) |  |
|  |  |  |  |  |  |  |  |  |  |
| FPN | Ref.^4^ | Ref.^4^ |  | Ref.^4^ | Ref.^4^ |  | Ref.^4^ | Ref.^4^ |  |
| SPN | 0.556 (0.23; 1.343) | 0.343 (0.082; 1.43) |  | 1.813 (0.568; 5.78) | 1.272 (0.324; 4.999) |  | 0.591 (0.209; 1.667) | 0.473 (0.096; 2.317) |  |
| **Alcoholic beverages per day** | | | | | | | | | |
|  | **<1 vs. 0** | **1+ vs. 0** |  | **<1 vs. 0** | **1+ vs. 0** |  | **<1 vs. 0** | **1+ vs. 0** |  |
| CO | Ref.^2^ | Ref.^2^ |  | Ref.^2^ | Ref.^2^ |  | Ref.^2^ | Ref.^2^ |  |
| FPN and SPN | 0.881 (0.459; 1.691) | **0.207 (0.064; 0.67)** |  | 0.808 (0.386; 1.691) | 0.523 (0.16; 1.709) |  | 0.808 (0.306; 2.138) | **0.121 (0.025; 0.574)** |  |
|  |  |  |  |  |  |  |  |  |  |
| FPN | Ref.^3^ | Ref.^3^ |  | Ref.^3^ | Ref.^3^ |  | Ref.^3^ | Ref.^3^ |  |
| SPN | **0.294 (0.111; 0.776)** | 0.218 (0.012; 3.983) |  | 0.858 (0.255; 2.889) | 1.072 (0.175; 6.585) |  | - ( -; -) | - ( - ; -) |  |

Table continues

**Supplementary Table 1:** Continued

| **Smoking status** | | | | | | | | | |
| --- | --- | --- | --- | --- | --- | --- | --- | --- | --- |
|  | **former vs. never** | **current vs. never** |  | **former vs. never** | **current vs. never** |  | **former vs. never** | **current vs. never** |  |
| CO | Ref.^4^ | Ref.^4^ |  | Ref.^4^ | Ref.^4^ |  | Ref.^4^ | Ref.^4^ |  |
| FPN and SPN | **0.249 (0.112; 0.554)** | **0.416 (0.179; 0.966)** |  | **0.346 (0.178; 0.675)** | **0.355 (0.159; 0.79)** |  | **0.106 (0.038; 0.298)** | **0.262 (0.114; 0.604)** |  |
|  |  |  |  |  |  |  |  |  |  |
| FPN | Ref.^5^ | Ref.^5^ |  | Ref.^5^ | Ref.^5^ |  | Ref.^5^ | Ref.^5^ |  |
| SPN | 1.951 (0.53; 7.18) | 1.642 (0.537; 5.024) |  | 0.584 (0.17; 2.002) | 0.75 (0.215; 2.617) |  | - ( -; -) | - ( - ; -) |  |
| **Pack years** | | | | | | | | | |
|  | **<5 vs. never smoked** | **5+ vs. never smoked** |  | **<5 vs. never smoked** | **5+ vs. never smoked** |  | **<5 vs. never smoked** | **5+ vs. never smoked** |  |
| CO | Ref.^4^ | Ref.^4^ |  | Ref.^4^ | Ref.^4^ |  | Ref.^4^ | Ref.^4^ |  |
| FPN and SPN | 0.773 (0.321; 1.859) | **0.149 (0.049; 0.459)** |  | 0.979 (0.427; 2.247) | **0.191 (0.077; 0.472)** |  | 0.58 (0.188; 1.793) | **0.078 (0.033; 0.185)** |  |
|  |  |  |  |  |  |  |  |  |  |
| FPN | Ref.^5^ | Ref.^5^ |  | Ref.^5^ | Ref.^5^ |  | Ref.^5^ | Ref.^5^ |  |
| SPN | 1.868 (0.509; 6.859) | 1.921 (0.48; 7.689) |  | 0.239 (0.037; 1.539) | 1.472 (0.431; 5.029) |  | - ( -; -) | - ( - ; -) |  |
| **Passive smoking** | | | | | | | | | |
|  | **yes vs. no** |  |  | **yes vs. no** |  |  | **yes vs. no** |  |  |
| CO | Ref.^6^ |  |  | Ref.^6^ |  |  | Ref.^6^ |  |  |
| FPN and SPN | **0.422 (0.212; 0.843)** |  |  | 0.463 (0.198; 1.08) |  |  | **0.336 (0.144; 0.784)** |  |  |
|  |  |  |  |  |  |  |  |  |  |
| FPN | Ref.^7^ |  |  | Ref.^7^ |  |  | Ref.^7^ |  |  |
| SPN | 0.284 (0.034; 2.383) |  |  | **3.826 (1.044; 14.02)** |  |  | 0.523 (0.085; 3.205) |  |  |

Adjustment variables were selected using directed acyclic graphs

Abbreviations: confidence interval (CI), cancer-free control (CO), first primary neoplasm (FPN), International Standard Classification for Education (ISCED), odds ratio (OR), second primary neoplasm (SPN)

^1^ Missing values are shown but not included in the analysis

^2^ Additionally adjusted for ISCED and ethnicity

^3^ Additionally adjusted for ISCED, ethnicity, and therapy of FPN

^4^ Additionally adjusted for ISCED

^5^ Additionally adjusted for ISCED and therapy of FPN

^6^ DAGs identified no additional adjustment variables for this model

^7^ Additionally adjusted for therapy of FPN
